# Supplementary figures and images for: Identification of a diguanylate cyclase expressed in the presence of plants and its application for discovering candidate gene products involved in plant colonization by Pantoea sp. YR343
Source: PLoS One. 2021 Jul 21;16(7):e0248607. doi: 10.1371/journal.pone.0248607 (PMC8294551; doi:10.1371/journal.pone.0248607)

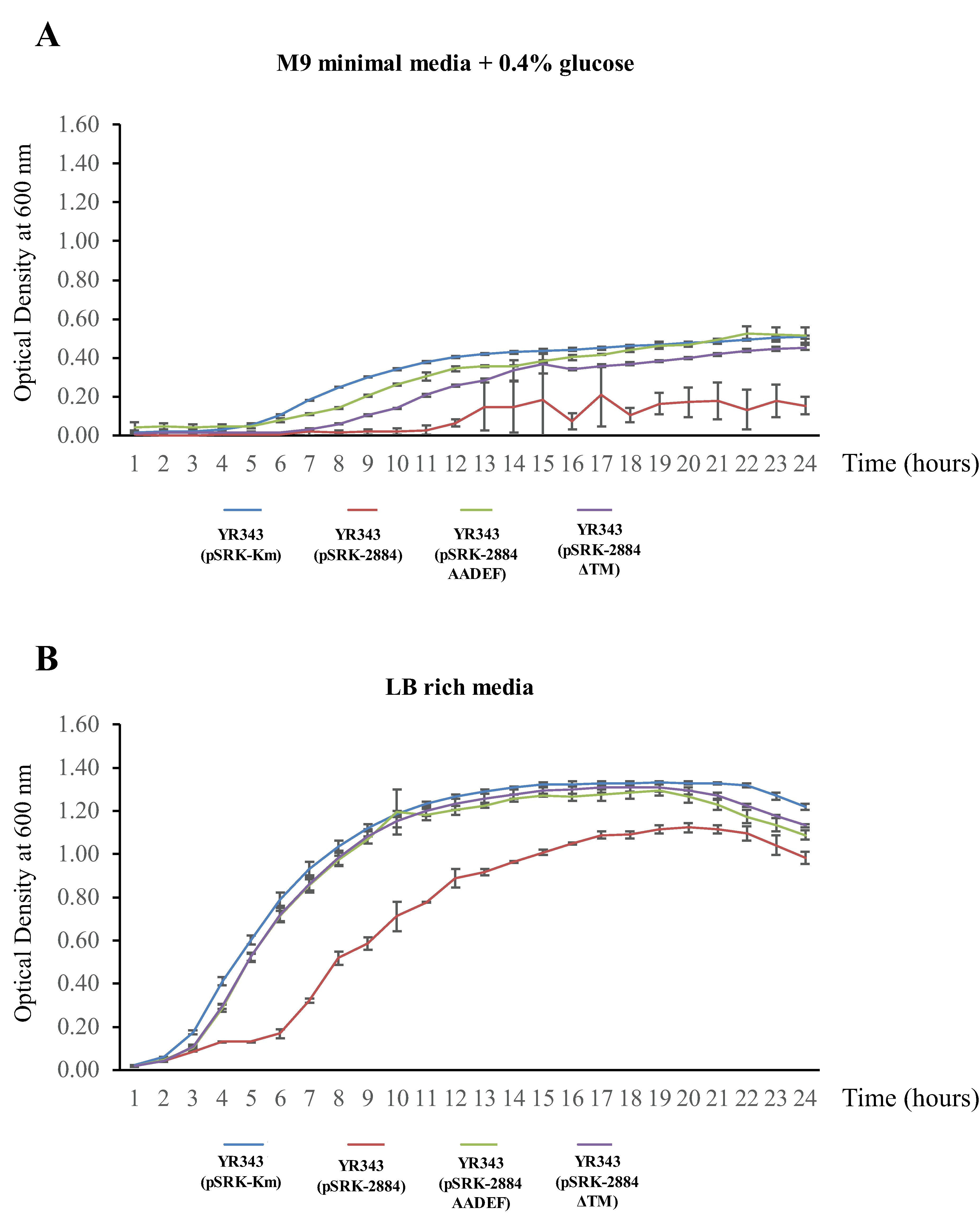

Supplement: S1 Fig — Growth curves of wild type (pSRK-Km) and the indicated DGC overexpressing strains in minimal media (A) and LB media (B). Error bars represent the standard deviation from three independent cultures. (TIF) [file pone.0248607.s001.tif]

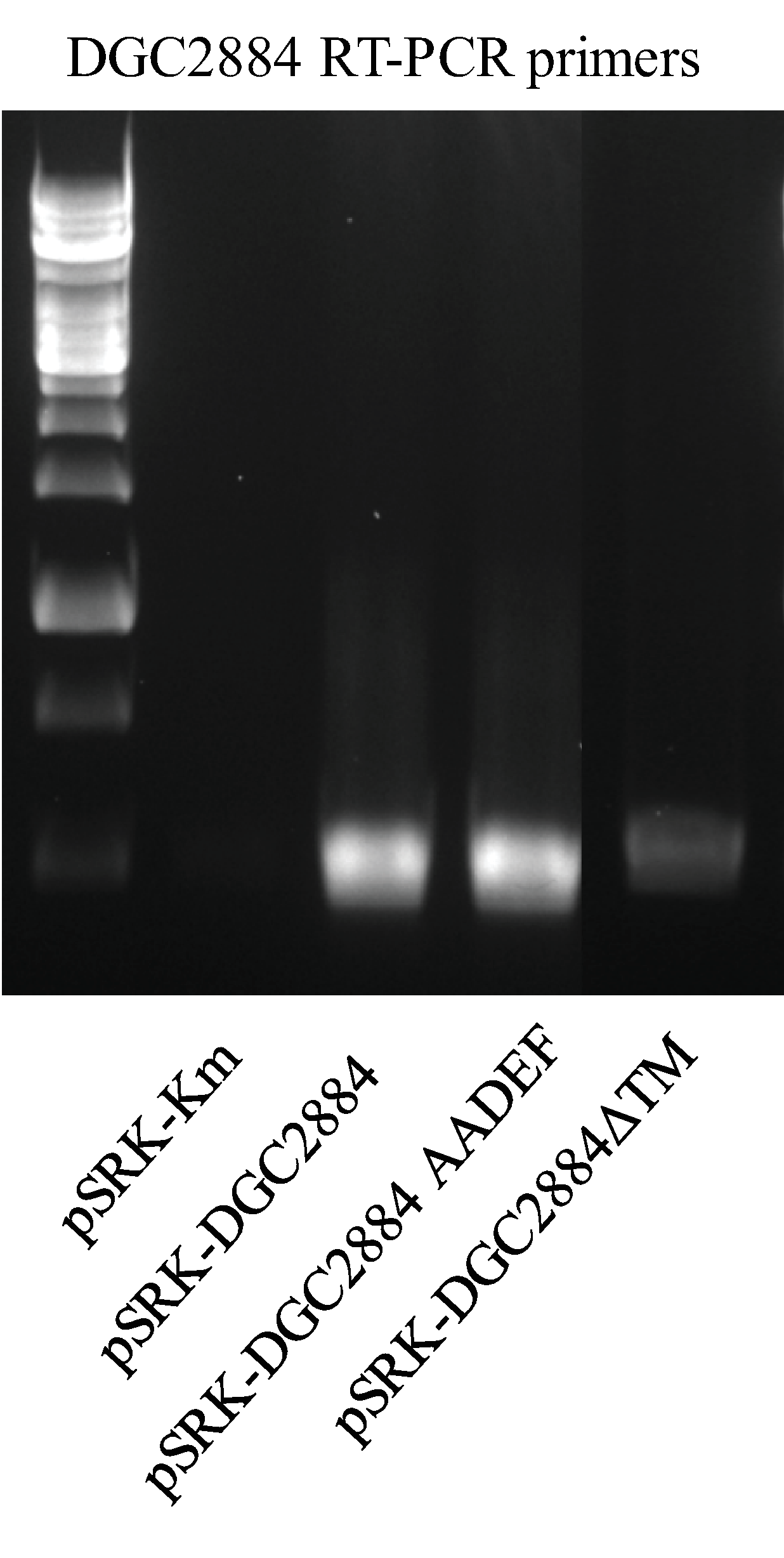

Supplement: S2 Fig — Image shown is representative of a minimum of 3 replicates. (TIF) [file pone.0248607.s002.tif]

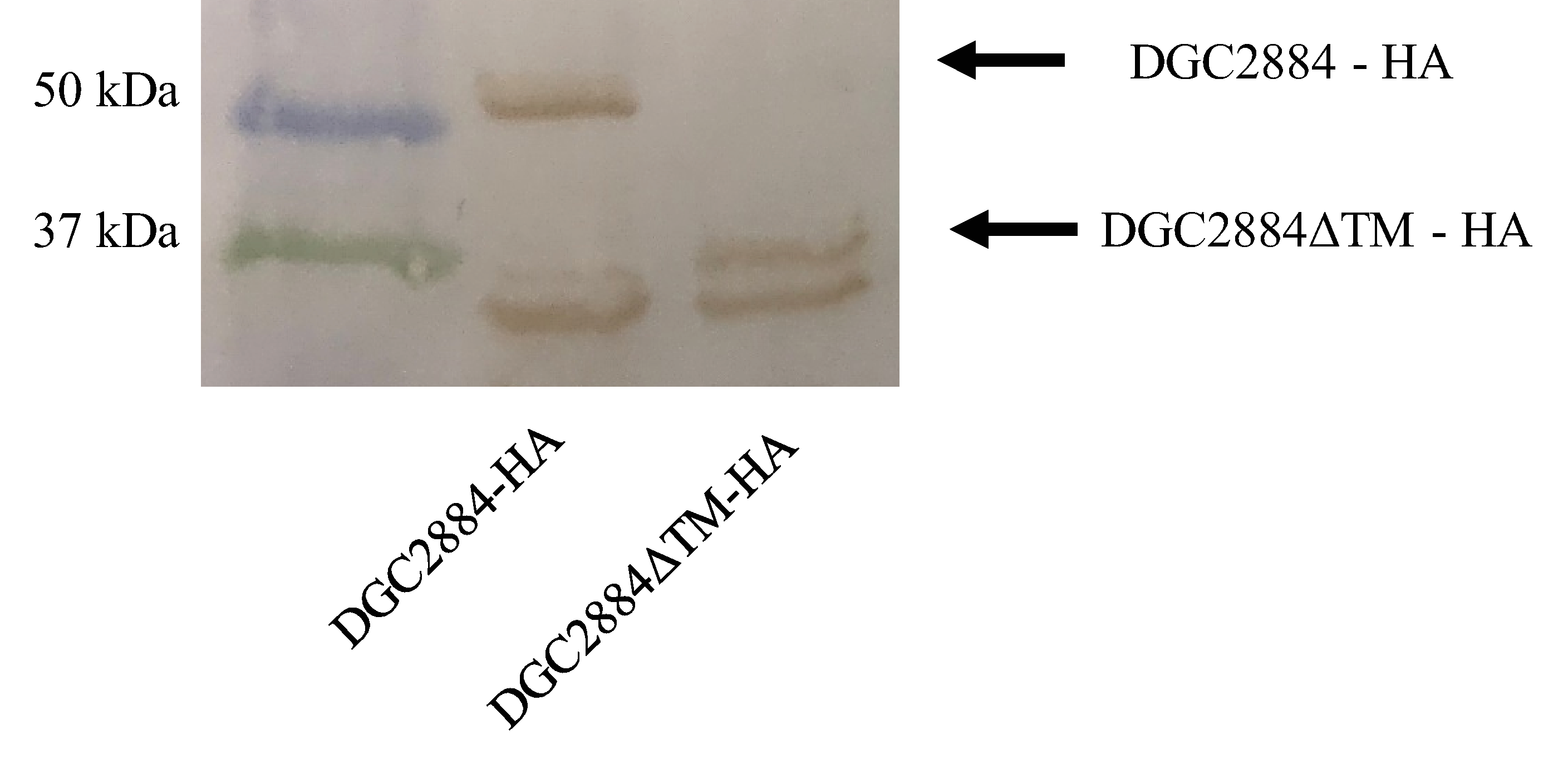

Supplement: S3 Fig — Weights of markers are indicated on the left and arrows point to bands that represent the indicated protein. (TIF) [file pone.0248607.s003.tif]

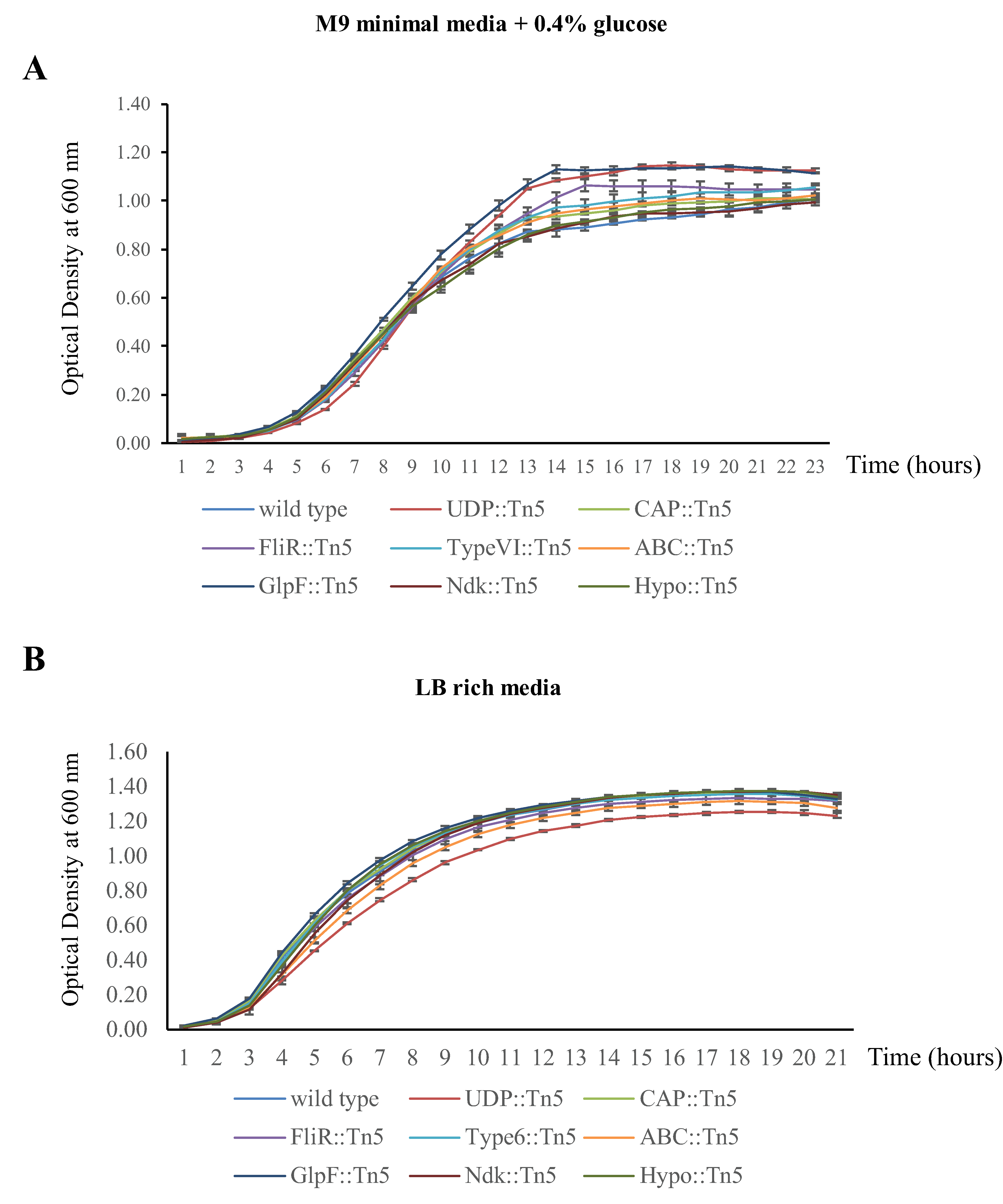

Supplement: S4 Fig — Growth curves of wild type Pantoea sp. YR343 and indicated transposon mutants in minimal media (A) and in LB media (B). Error bars represent the standard deviation from three independent cultures. (TIF) [file pone.0248607.s004.tif]

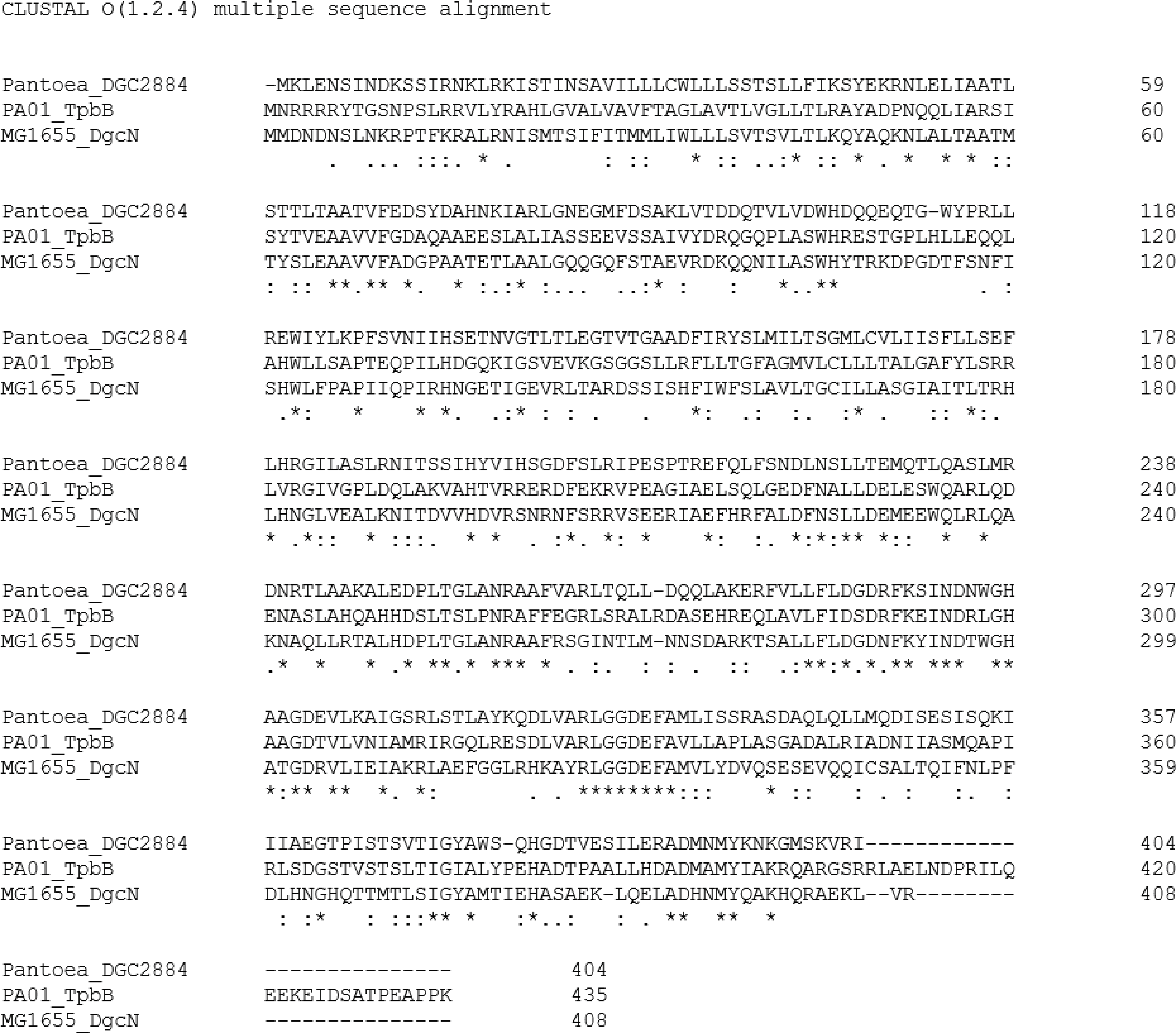

Supplement: S5 Fig — (TIF) [file pone.0248607.s005.tif]
