# Supplementary material for: Identification of a diguanylate cyclase expressed in the presence of plants and its application for discovering candidate gene products involved in plant colonization by Pantoea sp. YR343
Source: PLoS One. 2021 Jul 21;16(7):e0248607. doi: 10.1371/journal.pone.0248607 (PMC8294551; doi:10.1371/journal.pone.0248607)
Supplement: S1 Table — (PDF) [file pone.0248607.s006.pdf]

Supplementary Tables

TABLE S1. Strains and Plasmids used in this study

Strain or plasmid	Genotype or relevant characteristics	Reference or source
Plasmids		
pET DEST42	Expression vector	Invitrogen
pDEST14	Cloning vector	Invitrogen
pET DEST14-42	Expression vector with C-terminal V5 and His tags	this work
pET31b-Vc2 Spinach	Expression vector with Vc2-Spinach aptamer	(42)
pPROBE-NT	Cloning vector with promoter-less GFP (Km	(73)
pSRK-Km	Expression vector, (Km)	(74)
pSRK-Gm	Expression vector, (Gm*)	(74)
pRL27	Tn5-RL27 (Km^R-*ori*R6K) delivery vector	(76)
pRH016	pBBR1 gateway expression vector with C-terminal 3HA tag, (Cm*)	(75)
pRH018	pBBR1 gateway expression vector with C-terminal 13MYC tag, (Cm)	(75)
pPROBE-*DGC0366*	pPROBE containing promoter region of *DGC0366*	this work
pPROBE-*DGC0751*	pPROBE containing promoter region of *DGC0751*	this work
pPROBE-*DGC0995*	pPROBE containing promoter region of *DGC0995*	this work
pPROBE-*DGC1008*	pPROBE containing promoter region of *DGC1008*	this work
pPROBE-*DGC1023*	pPROBE containing promoter region of *DGC1023*	this work
pPROBE-*DGC1024*	pPROBE containing promoter region of *DGC1024*	this work
pPROBE-*DGC1089*	pPROBE containing promoter region of *DGC1089*	this work
pPROBE-*DGC1854*	pPROBE containing promoter region of *DGC1854*	this work
pPROBE-*DGC2196*	pPROBE containing promoter region of *DGC2196*	this work
pPROBE-*DGC2242*	pPROBE containing promoter region of *DGC2242*	this work
pPROBE-*DGC2465*	pPROBE containing promoter region of *DGC2465*	this work
pPROBE-*DGC2697*	pPROBE containing promoter region of *DGC2697*	this work
pPROBE-*DGC2884*	pPROBE containing promoter region of *DGC2884*	this work
pPROBE-*DGC3006*	pPROBE containing promoter region of *DGC3006*	this work
pPROBE-*DGC3134*	pPROBE containing promoter region of *DGC3134*	this work
pPROBE-*DGC3217*	pPROBE containing promoter region of *DGC3217*	this work
pPROBE-*DGC3247*	pPROBE containing promoter region of *DGC3247*	this work
pPROBE-*DGC3482*	pPROBE containing promoter region of *DGC3482*	this work

pPROBE-*DGC3621*	pPROBE containing promoter region of *DGC3621*	this work
pPROBE-*DGC4070*	pPROBE containing promoter region of *DGC4070*	this work
pSRK (Km)-*DGC2884*	pSRK-Km containing full gene sequence of *DGC2884*	this work
pSRK (Km)-*DGC2884 AADEF*	pSRK-Km containing gene sequence of *DGC2884 AADEF*	this work
pSRK (Km)-*DGC2884ΔTM*	pSRK-Km containing full gene sequence of *DGC2884ΔTM*	this work
pRH016-*DGC2884*	pRH016 containing *DGC2884* with C-terminal 3HA tag	this work
pRH016-*DGC2884ΔTM*	pRH016 containing *DGC2884ΔTM* with C-terminal 3HA tag	this work
pRH018-*DGC2884ΔTM*	pRH018 containing *DGC2884ΔTM* with C-terminal 13MYC tag	this work
pBT270	pUC18-miniTn7T2-PA1/04/03-GFP (Ap*, Gm)	gift from Dr. B. S. Tseng
pBT277	pUC18-miniTn7T2-PA1/04/03-mCherry (Ap, Gm)	(78)
Strains		
Escherichia co		
TOP10	General cloning strain	ThermoFisher Scientific
EA145	Mating strain containing pRL27, DAP auxotroph	gift from Dr. A. Buchan
BL21 DE3 Star	Protein expression strain	ThermoFisher Scientific
Pantoea sp.		
YR343	wild type strain	
YR343 (pPROBE-*DGC0366*)	YR343 containing pPROBE-*DGC0366*	this work
YR343 (pPROBE-*DGC0751*)	YR343 containing pPROBE-*DGC0751*	this work
YR343 (pPROBE-*DGC0995*)	YR343 containing pPROBE-*DGC0995*	this work
YR343 (pPROBE-*DGC1008*)	YR343 containing pPROBE-*DGC1008*	this work
YR343 (pPROBE-*DGC1023*)	YR343 containing pPROBE-*DGC1023*	this work
YR343 (pPROBE-*DGC1024*)	YR343 containing pPROBE-*DGC1024*	this work
YR343 (pPROBE-*DGC1089*)	YR343 containing pPROBE-*DGC1089*	this work
YR343 (pPROBE-*DGC1854*)	YR343 containing pPROBE-*DGC1854*	this work
YR343 (pPROBE-*DGC2196*)	YR343 containing pPROBE-*DGC2196*	this work
YR343 (pPROBE-*DGC2242*)	YR343 containing pPROBE-*DGC2242*	this work
YR343 (pPROBE-*DGC2465*)	YR343 containing pPROBE-*DGC2465*	this work
YR343 (pPROBE-*DGC2697*)	YR343 containing pPROBE-*DGC2697*	this work
YR343 (pPROBE-*DGC2884*)	YR343 containing pPROBE-*DGC2884*	this work
YR343 (pPROBE-*DGC3006*)	YR343 containing pPROBE-*DGC3006*	this work
YR343 (pPROBE-*DGC3134*)	YR343 containing pPROBE-*DGC3134*	this work
YR343 (pPROBE-*DGC3217*)	YR343 containing pPROBE-*DGC3217*	this work
YR343 (pPROBE-*DGC3247*)	YR343 containing pPROBE-*DGC3247*	this work
YR343 (pPROBE-*DGC3482*)	YR343 containing pPROBE-*DGC3482*	this work

YR343 (pPROBE-*DGC3621*)	YR343 containing pPROBE-*DGC3621*	this work
YR343 (pPROBE-*DGC4070*)	YR343 containing pPROBE-*DGC4070*	this work
YR343 (pSRK-Km)	YR343 containing pSRK-Km	this work
YR343 (pSRK-*DGC2884*)	YR343 containing pSRK-*DGC2884* (Km)	this work
YR343 (pSRK-*DGC2884 AADEF*)	YR343 containing pSRK-*DGC2884* AADEF (Km)	this work
YR343 (pSRK-*DGC2884ΔTM*)	YR343 containing pSRK-*DGC2884ΔTM* (Km)	this work
YR343 (pSRK(Gm)-*DGC2884*)	YR343 containing pSRK(Gm)-*DGC2884* (Gm)	this work
BL21 DE3 Star (pET31b-Vc2 Spinach + pSRK-Km)	BL21 DE3 Star containing pET31b-Vc2 Spinach (Cb) and pSRK-Km	this work
BL21 DE3 Star (pET31b-Vc2 Spinach + pSRK-*DGC2884*)	BL21 DE3 Star containing pET31b-Vc2 Spinach (Cb) and pSRK-*DGC2884* (Km)	this work
BL21 DE3 Star (pET31b-Vc2 Spinach + pSRK-*DGC2884ΔTM*)	BL21 DE3 Star containing pET31b-Vc2 Spinach (Cb) and pSRK-*DGC2884ΔTM* (Km)	this work
BL21 DE3 Star (pET31b-Vc2 Spinach + *pSRK-DGC2884 AADEF*)	BL21 DE3 Star containing pET31b-Vc2 Spinach (Cb) and pSRK-*DGC2884 AADEF*(Km)	this work
YR343 (pRH016-*DGC2884*)	YR343 containing pRH016-*DGC2884* (Cm)	this work
YR343 (pRH018-*DGC2884ΔTM*)	YR343 containing pRH018-*DGC2884ΔTM* (Cm)	this work
YR343 (pRH018-*ipdC*)	YR343 containing pRH018-*ipdC* (Cm)	Morrell-Falvey Lab
YR343:GFP	YR343 with GFP integrated chromosomally via pBT270	this work
CAP::Tn5	YR343 mutant with a transposon insertion in PMI39_03059	this work
CAP:mCherry	CAP::Tn5 with mCherry integrated chromosomally via pBT277	this work
UDP::Tn5	YR343 mutant with a transposon insertion in PMI39_01848	this work
UDP:mCherry	UDP::Tn5 with mCherry integrated chromosomally via pBT277	this work
FliR::Tn5	YR343 mutant with a transposon insertion in PMI39_02188	this work
TypeVI::Tn5	YR343 mutant with a transposon insertion in PMI39_03162	this work
GlpF::Tn5	YR343 mutant with a transposon insertion in PMI39_04394	this work
ABC::Tn5	YR343 mutant with a transposon insertion in PMI39_04218	this work
Ndk::Tn5	YR343 mutant with a transposon insertion in PMI39_03579	this work
Hypo::Tn5	YR343 mutant with a transposon insertion in PMI39_03065	this work

*Antibiotic resistance: Km, kanamycin; Gm, gentamycin; Ap, ampicillin; Cm, chloramphenicol
