# Supplementary material for: Identification of a diguanylate cyclase expressed in the presence of plants and its application for discovering candidate gene products involved in plant colonization by Pantoea sp. YR343
Source: PLoS One. 2021 Jul 21;16(7):e0248607. doi: 10.1371/journal.pone.0248607 (PMC8294551; doi:10.1371/journal.pone.0248607)
Supplement: S2 Table — (PDF) [file pone.0248607.s007.pdf]

TABLE S2. Primers used in this study

Primer Name	Primer Sequence (5’ -> 3’)
DGC0366 prom-For	CGCGGATCCCCGTAGCAAAGTCAGGCC
DGC0366 prom-Rev	CCGGAATTCCCCTGTGCCTGCATCACT
DGC0751 prom-For	CGCGGATCCCGATGTGACTACCGAATG
DGC0751 prom-Rev	CCGGAATTCGTTGTCCTCTGTTTTCAA
DGC0995 prom-For	CGCGGATCCCTGCGCTACTTCAACAGC
DGC0995 prom-Rev	CCGGAATTCCACGATGCCATTTCCGCC
DGC1008 prom-For	CGCGGATCCCGTCAACGCATGATGATT
DGC1008 prom-Rev	CCGGAATTCTGTTATTCGCCTATTGCT
DGC1023 prom-For	CGCGGATCCTGGCGTTTAGCGATAACG
DGC1023 prom-Rev	CCGGAATTCAGTGATTTCCCAAGTAAA
DGC1024 prom-For	CGCGGATCCTTTCGCTTAACGACTGAC
DGC1024 prom-Rev	CCGGAATTCGTCCGCTCCTAAATTCCA
DGC1089 prom-For	CGCGGATCCATCCTTTGTCTCTGGTGT
DGC1089 prom-Rev	CCGGAATTCACGTCTGAACCCTGTAAC
DGC1854 prom-For	CGCGGATCCGAAAAGCCCTATACCGCG
DGC1854 prom-Rev	CCGGAATTCGCAAGAATCCAGCTGCGC
DGC2196 prom-For	CGCGGATCCTGAGGCGTTCCACAGTGA
DGC2196 prom-Rev	CCGGAATTCGTGCATCCCTGCTTCGAA
DGC2242 prom-For	CGCGGATCCCGGATGAATTTGCTTTAG
DGC2242 prom-Rev	CCGGAATTCTTTAAGGTGAGCCTGACA
DGC2334 prom-For	CGCGGATCCAGGTGTTGGCGCGCAAGC
DGC2334 prom-Rev	CCGGAATTCAACTTCTCCAGGCCACAT
DGC2465 prom-For	CGCGGATCCGCGCATAGTAGCAACGCC
DGC2465 prom-Rev	CCGGAATTCGCGCAATGCTCGCGAAAT
DGC2697 prom-For	CGCGGATCCGCTTTCCCAGCCAGGCCG
DGC2697 prom-Rev	CCGGAATTCGGAAACTTCCTCCGGGGG
DGC2884 prom-For	CGCGGATCCGTTAAATCACTTCAAGGG
DGC2884 prom-Rev	CCGGAATTCCTTATTTGCTTCCATTGC
DGC3006 prom-For	CGCGGATCCGGCTGGCACTTAAGTAAG
DGC3006 prom-Rev	CCGGAATTCGGTCGGCTGATGGAGAGG
DGC3134 prom-For	CGCGGATCCATCCAAAATGAAACTTTA
DGC3134 prom-Rev	CCGGAATTCGTGAAAACCTCAAAAGAG
DGC3217 prom-For	CGCGGATCCCTGTCCTAAACCTGACTC
DGC3217 prom-Rev	CCGGAATTCAGTGGTCGGAGCTCTTGA

DGC3247 prom-For	CGCGGATCCACAATACTTCTCATCTTG
DGC3247 prom-Rev	CCGGAATTCTATTATTCTCGTGACAGC
DGC3482 prom-For	CGCGGATCCCCGGTGCCGATCTCATTT
DGC3482 prom-Rev	CCGGAATTCGCGGTTACTCTTATTAAT
DGC3621 prom-For	CGCGGATCCGGCCATTTTACGACGCCA
DGC3621 prom-Rev	CCGGAATTCAACGCGCCGGCCTTAGTG
DGC4070 prom-For	CGCGGATCCGCAATTCCGCTTGCAGGG
DGC4070 prom-Rev	CCGGAATTCGCACGGGAAGTATCAGGA
DGC2884_AADEF For	CTG GTC GCC CGA TTA **GCC GCC** GAT GAG TTT GCC ATG
DGC 2884_AADEF Rev	CAT GGC AAA CTC ATC **GGC GGC** TAA TCG GGC GAC CAG
DGC2884 For (BamHI)	CGCGGATCCATGAAATTAGAAAATTCAATCAAC
DGC2884_noTM For (XbaI)	GC TCT AGA ATG AGT GAG TTT CTC CAT CGC
DGC2884 Rev (HindIII)	CCCAAGCTTTCATATCCGCACCTTACTCATTCC
GWdgc2884 For	CACCGTTAAATCACTTCAAGGG
GW DGC2884_noTM For	CACC ATG AGT GAG TTT CTC CAT CGC
GWdgc2884 Rev	TATCCGCACCTTACTCATTCC

Underlined portions of primer sequences correspond to restriction enzyme sites: BamHI (For) and EcoRI or HindIII (Rev). Bold letters represent sites targeted for site-directed mutagenesis.
