# Supplementary material for: Identification of a diguanylate cyclase expressed in the presence of plants and its application for discovering candidate gene products involved in plant colonization by Pantoea sp. YR343
Source: PLoS One. 2021 Jul 21;16(7):e0248607. doi: 10.1371/journal.pone.0248607 (PMC8294551; doi:10.1371/journal.pone.0248607)

Original Gel Image

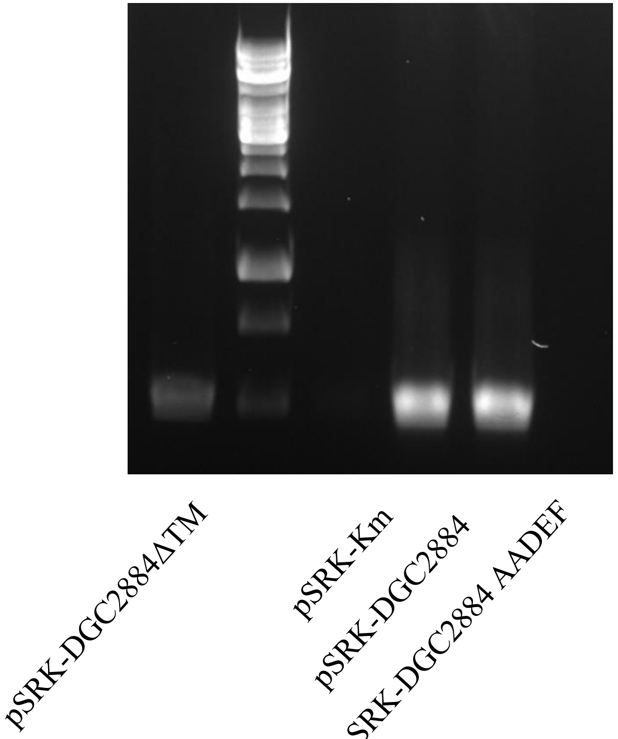

Original unedited western blot (3 sets)

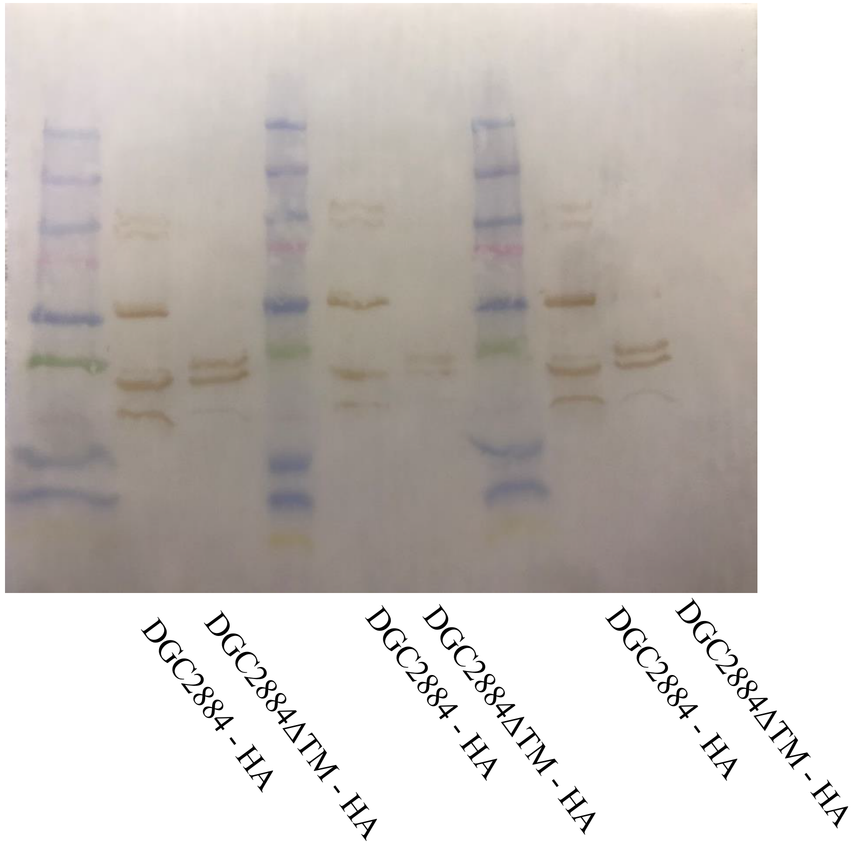

Supplement: S1 Raw images — (PDF) [file pone.0248607.s008.pdf]
